# Influences of Polyphenols on the Properties of Crosslinked Acellular Fish Swim Bladders: Experiments and Molecular Dynamic Simulations

**DOI:** 10.3390/polym16081111

**Published:** 2024-04-16

**Authors:** Yuqing Han, Jie Jiang, Jinjin Li, Ling Zhao, Zhenhao Xi

**Affiliations:** 1 State Key Laboratory of Chemical Engineering, School of Chemical Engineering, East China University of Science and Technology, Shanghai 200237, China; hanyuqing0729@163.com (Y.H.); jiangjie@ecust.edu.cn (J.J.); zhaoling@ecust.edu.cn (L.Z.); 2 Shanghai Key Laboratory of Multiphase Materials Chemical Engineering, East China University of Science and Technology, Shanghai 200237, China

**Keywords:** polyphenols, acellular fish swim bladder, crosslinking, molecular dynamic simulation, enzymic stability

## Abstract

Acellular fish swim bladders (AFSBs) are a promising biomaterial in tissue engineering, however, they may suffer from rapid degradation due to enzyme invasion. In this work, natural polyphenols, including epigallocatechin gallate (EGCG), proanthocyanidin (PC), tannic acid (TA) and protocatechuic acid (PCA), were utilized to improve the properties of AFSBs through crosslinking modifications. Fourier transform infrared (FTIR) results indicate that the triple helix of the collagen in AFSBs is well preserved after crosslinking. The differential scanning calorimetry (DSC), water contact angle (WCA) and in vitro degradation tests indicate that the polyphenol-crosslinked AFSBs exhibit improved thermal stability, enzymatic stability, hydrophilicity and mechanical properties. Among them, EGCG with multiple phenolic hydroxyl groups and low potential resistance is more favorable for the improvement of the mechanical properties and enzymatic stability of AFSBs, as well as their biocompatibility and integrity with the collagen triple helix. Moreover, the crosslinking mechanism was demonstrated to be due to the hydrogen bonds between polyphenols and AFSBs, and was affected by the molecular size, molecular weight and the hydroxyl groups activity of polyphenol molecules, as clarified by molecular dynamic (MD) simulations. The approach presented in this work paves a path for improving the properties of collagen materials.

## 1. Introduction

In the last decade, the use of aquatic collagen-based materials has become prevalent in tissue engineering due to concerns about the outbreak of bovine spongiform encephalopathy and transmissible spongiform encephalopathy. Fish swim bladders (FSBs) are byproducts of fish and mainly consist of fibrillar, type I collagen and multilayered transitional epithelial cells [1]. Acellular fish swim bladders (AFSBs), which have low zoonotic risks, extraordinary biocompatibility and fewer religious restrictions, are obtained through decellularization and have been used in tissue engineering as scaffolds. Li et al. [2] confirmed that AFSB utilized as a dura mater substitute can meet the demands of clinical applications. Liu et al. [3] prepared AFSB-based scaffolds crosslinked by glutaraldehyde (GA) for cardiovascular diseases, which showed superior anti-calcification properties and biocompatibility compared to bovine pericardium, revealing their promising application for use in cardiovascular biomaterials [4,5,6]. Furthermore, AFSBs have also shown potential for use in several fields, such as wound healing [7], cervical oesophagoplasty [8] and cystoplasty [9]. Considering the advantages and microstructure of AFSBs, they could be a favorable substitute in regenerative medicine. However, AFSBs are prone to losing their structural integrity and desirable mechanical properties during the implantation period due to the high enzymatic turnover rate of extracellular matrix (ECM) proteins in the body [10].

Crosslinking has proven to be an effective method for improving the mechanical strength and biodegradation of collagen-based membranes [11,12]. Physical crosslinking, chemical crosslinking [13,14] and enzymatic crosslinking [15] are the primary crosslinking methods. Chemical crosslinking has been confirmed to be the most effective approach, using chemical crosslinking agents such as GA, 1-ethyl-3-(3-dimethylaminopropyl) carbodiimide hydrochloride (EDC)/N-hydroxysuccinimide (NHS), genipin and polyphenols, since it is easy to operate and has an effective crosslinking effect. GA is the most widely used chemical crosslinking agent and can significantly improve the mechanical properties of collagen membranes. However, the potential cytotoxicity and inflammatory response of GA limits the extent of tissue integration in implanted materials [16]. 

Polyphenols are a class of natural compound mainly derived from plants with abundant hydroxyl groups, and can crosslink with macromolecules such as proteins and polysaccharides through hydrogen bonds (H-bonds) [17]. As reported, polyphenols can form H-bonds with the hydroxyl, carboxyl, amino and amide groups of the side chains of collagen. H-bonds play an important role in the stabilization of collagen by natural polyphenols, and different dipole characters of the individual phenolic substances appear to influence their bonding to collagen [18]. Furthermore, the connective tissues are protected from the degrading of elastases since the polyphenols can bond to elastin to inhibit the enzymatic degradation [19]. Procyanidin (PC) is the most commonly used polyphenols for the modification of collagen membranes [18] and heart valves [20], and inhibit the osteodifferentiation and calcification of valvular-related cells. The research by Zhai et al. [21] shows that PC can crosslink porcine heart valves effectively without toxicity and this might be a useful approach for the preparation of bioprosthetic heart valves. In addition, pericardium matrices crosslinked by quercetin [22] and curcumin [23] were prepared and showed an ability to protect ECMs from the deposition of minerals, as well as dramatically reduce Ca^2+^ content in simulated body fluid. Polyphenols extracted from Nebbiolo grape pomace [24], the fruit of Terminalia chebula Retz [25] and pomegranate pericarp [26] proved to contribute to the enhancement of collagen membranes in mechanical properties, antibacterial activity, fibroblast cell proliferation and resistance to hydrolysis by collagenase. Wu et al. [27] revealed the mechanism of crosslinking between collagen and polyphenols with complex molecular structures under unoxidized and oxidized conditions in vitro. Polyphenols tend to approach the surfaces of protein molecules through hydrophobic bonds and enter a hydrophobic bag, which subsequently generates multipoint hydrogen bonding [28]. Nonetheless, the crosslinking mechanism of polyphenols and AFSBs seems to be different from collagen solutions due to the microstructure and complex components of AFSBs, thus, it needs to be further explored. 

**Figure 1:**
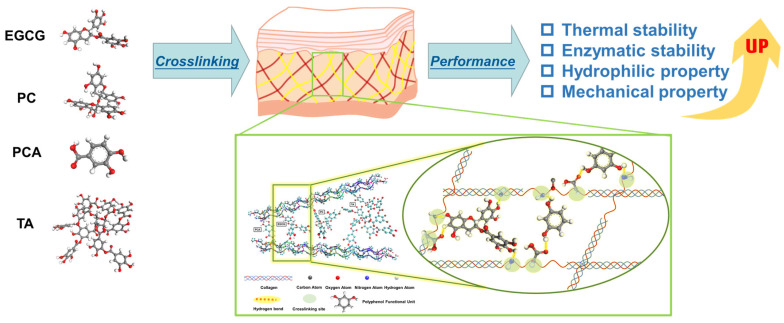
Schematic illustration of the experimental process and crosslinking mechanism between polyphenols and acellular fish swim bladders (AFSBs).

In this work, AFSBs were respectively crosslinked with various polyphenols, i.e., epigallocatechin gallate (EGCG), tannic acid (TA), PC and protocatechuic acid (PCA), to improve their properties. The effects of the molecular structure of polyphenols on the thermal stability, hydrophilicity, enzymatic stability, mechanical property and biocompatibility of the crosslinked AFSBs were investigated in detail. In addition, the interaction mechanism regarding the H-bonds formed between polyphenols and collagen was investigated by molecular dynamic (MD) simulations. Figure 1 illustrates the structures of polyphenols and the crosslinking mechanism between polyphenols and AFSBs.

## 2. Materials and Methods

### 2.1. Materials

FSBs of silver carp were purchased from a local aquatic product market (Shanghai, China). Sodium dodecyl sulfate (SDS) was purchased from Shanghai Macklin Biochemical Co. (Shanghai, China). Triton X-100 was obtained from Shanghai Titan Scientific Co. (Shanghai, China). DNase and RNase were purchased from Shanghai Yuanye Bio-Technology Co. (Shanghai, China). EGCG, PC, PCA and TA were obtained from Shanghai Macklin Biochemical Co. (Shanghai, China). The cell proliferation and cytotoxicity assay kit (CCK-8 kit) and fetal bovine serum (FBS) were obtained from Beyotime Biotech (Shanghai, China). The aforementioned chemicals were used as received without further purification.

### 2.2. Preparation and Crosslinking of AFSBs

The FSBs were washed with cold phosphate-buffered saline (PBS). After removing the connected mucous membranes and tissues from the FSBs, AFSBs were obtained according to a previously reported method [3]. Briefly, FSBs were treated with SDS aqueous (0.5% *w*/*v*) and Triton X-100 aqueous (1% *w*/*v*) for 6 h and 0.5 h at room temperature, respectively. Then, the tissues were washed with PBS three times and immersed in PBS containing DNase (100 units/mL) and RNase (5 units/mL) overnight at 37 °C. After washing with PBS thoroughly, the AFSBs were stored at −20 °C until further treatment.

As for modification, the AFSBs were crosslinked by soaking in different polyphenol solutions containing the same amount of hydroxyl groups at 37 °C for 48 h. The polyphenol solutions with 1 mmol hydroxyl groups were prepared by individually dissolving 57 mg EGCG, 59 mg PC, 68 mg TA and 77 mg PCA in 50 mL de-ionized (DI) water. Additionally, the AFSBs were crosslinked using 50 mL GA solution with 1 mmol aldehyde groups under the same conditions as the control. The AFSBs treated with EGCG, PC, TA, PCA and GA were defined as EGCG, PC, TA, PCA and GA, respectively, while the uncrosslinked AFSB was defined as UN.

The influences of polyphenols on the properties of crosslinked AFSBs were explored through experiments, and the relevant characterization method details are described in the Supporting Information.

### 2.3. Simulation Details

In order to further detect the interactions between collagen and polyphenols, MD simulations were carried out using GROMACS 5.0.7 [29] with the Gromos54a7 force field [30]. The collagen molecule was downloaded from the protein data bank (PDB) archive and the structures of EGCG, PC, TA and PCA were optimized using the B3LYP/6-11(G) method. The force field parameters of polyphenols were obtained from the Automated Topology Builder (ATB) [31], and the H_2_O molecule was described by the SPC/E model [32]. Collagen molecules, polyphenols molecules and H_2_O molecules were packed in a cubic box (6.0 × 6.0 × 6.0 nm^3^) using Packmol 18.169 software [33], and the periodic boundary condition was utilized in all directions. The collagen molecules were fixed in the same position in the initial configuration of each system. The compositions of the collagen/polyphenols systems are listed in Table S1 and the number of hydroxyl groups in each system was controlled to be the same. 

The simulations were carried out as follows. The energy of the simulation system was minimized using a steep integrator for 5000 steps, followed by a 500 ps NVT and a 20 ns NPT with a time step of 1 fs. The Berendsen thermostat was used to control the temperature at 300 K and pressure at 1 bar. An accurate leap-frog stochastic dynamics integrator and the particle mesh Ewald method (PME) were employed with a cut-off distance of 1.2 nm for all simulations, in order to treat the long-range electrostatic interactions. Furthermore, the bond-length distances were constrained by the Linear Constraint Solver (LINCS) algorithm. The Lennard-Jones interaction was used to describe the van der Waals interaction, where the cut-off distance was set to 1.2 nm. All the snapshots were displayed through the VMD 1.9.1 software [34].

## 3. Results and Discussion 

### 3.1. Structural Characterization of AFSBs

**Figure 2:**
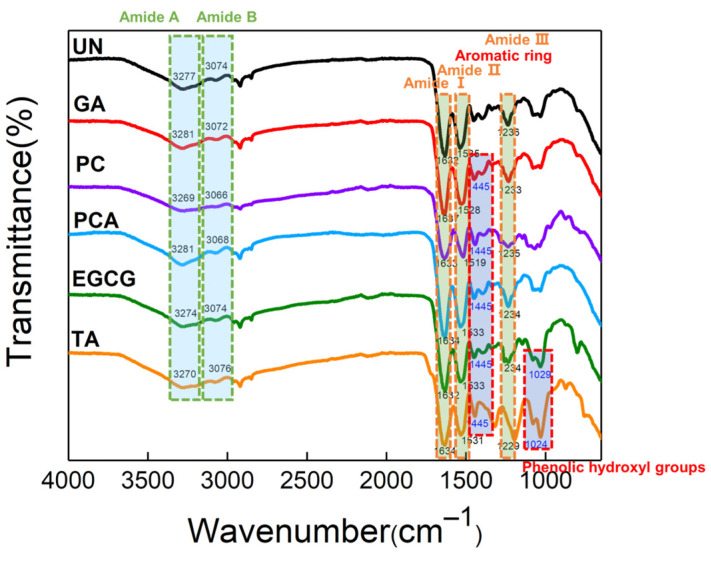
Attenuated total reflection Fourier transformed infrared (ATR-FTIR) spectra of different polyphenol-crosslinked AFSBs.

In order to evaluate the functional groups of polyphenols and the structural integrity of the crosslinked AFSBs, attenuated total reflection Fourier transformed infrared (ATR-FTIR) spectroscopy was applied and the results are shown in Figure 2. The spectra of protein molecules are correlated directly to their backbone conformation [35], and the intact triple helix conformation of collagen can be characterized by typical amide bands in the spectra [36]. The spectra present typical absorption of protein molecules. The characteristic absorption at 3277 cm^−1^ and 3074 cm^−1^ are mainly associated with the stretching vibrations of N-H groups of amide A and B bands [37,38], respectively. In particular, amide Ⅰ at 1632 cm^−1^ implies the stretching vibrations of carbonyl (C=O), amide Ⅱ centered at 1535 cm^−1^ is assigned to the N-H bending vibrations and C-N stretching vibrations, and amide Ⅲ absorbance at 1236 cm^−1^ arises from the C-N stretching and N-H bending vibrations from amide linkages, as well as CH_2_ groups bending vibrations in the glycine backbone and proline side chains [39]. There was no significant change or shift in the position of the main amide bands of crosslinked AFSBs compared with the UN, especially the amide I band, which is correlated to the helix structure of collagen [40], suggesting that the triple helix can be preserved integrally after crosslinking by polyphenols. 

Additional absorption was observed in the range of 1000 cm^−1^ to 1455 cm^−1^ in crosslinked AFSBs due to the introduction of polyphenols and their bonding to collagen. For instance, the enhanced absorption at 1445 cm^−1^ in EGCG and TA was ascribed to the aromatic ring stretching vibrations, and the absorption at 1029 cm^−1^ was associated with the phenolic hydroxyl groups of polyphenols. The expanded absorption of amide A band, to some weak extent, can be observed in the EGCG and TA groups, indicating H-bonds between polyphenols and collagen [41]. However, it is difficult to observe the expanded bands in other groups due to the masking effect of the intense absorption of amide bands. 

**Figure 3:**
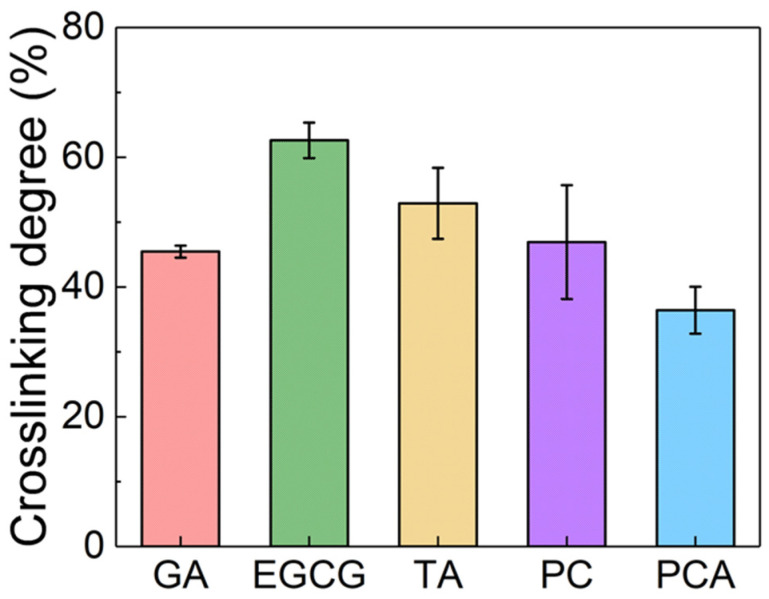
Crosslinking degrees of crosslinked AFSBs.

The crosslinking degree was determined by calculating the free content of amine using a ninhydrin assay, where ninhydrin can react with free amine groups in protein fibers to produce purple products, which can be detected by absorbance. The results are shown in Figure 3, and the experimental diagrams are presented in Figure S1. The crosslinking degree of EGCG, TA and PC was 62.6%, 52.9% and 46.9%, respectively, which was higher than that of GA (45.5%). PCA had the lowest crosslinking degree at 36.4%. The free amino groups of protein fibers in AFSBs as hydrogen donors can form H-bonds with phenolic hydroxyl groups in the polyphenols as hydrogen receptors [42], while phenolic hydroxyl groups can also form H-bonds as hydrogen donors with carbonyl of protein fibers [43]. The higher crosslinking degree of EGCG-crosslinked AFSB is associated with the higher phenolic hydroxyl activity and stronger interactions with protein fibers of EGCG compared to other polyphenols. Actually, the interaction of polyphenols with proteins through H-bonds and hydrophobicity is not as effective as the covalent bonds formed by GA, resulting in a lower crosslinking degree for PCA than GA, despite their similar molecular weights. Crosslinking increased the mechanical stability of the materials: the higher the crosslinking degree, the better the mechanical strength of the samples. 

### 3.2. Surface Wettability and Swelling Behavior

**Figure 4:**
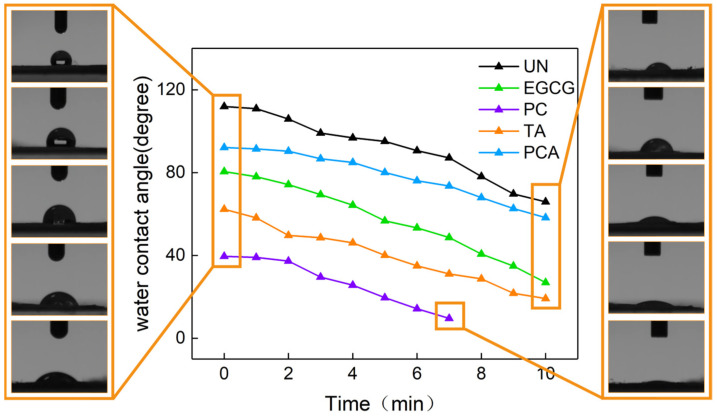
Water contact angles (WCAs) of polyphenol-crosslinked AFSBs as a function of time.

The water contact angle (WCA) test was performed in order to detect the hydrophilic properties of crosslinked AFSBs, and the results are shown in Figure 4. The WCA of the UN was about 112°, showing the structural integrity of the collagen and the dense surface of the AFSB. The introduction of the polyphenols reduced the WCA and improved the hydrophilicity of the AFSBs, which can facilitate cell growth and adhesion. Among them, the WCAs of PC and TA were decreased to 40° and 62°, respectively, showing the greatest improvement in hydrophilicity. Meanwhile, PCA and EGCG showed relatively weak improvements in hydrophilicity; their WCAs were reduced by 20° and 30°, respectively. Hydrophilic improvement is the comprehensive result of the introduction of hydroxyl groups through polyphenols and the reduction of hydrophilic groups in collagen [27]. The residual hydroxyl groups will increase the hydrophilicity of crosslinked AFSBs if the introduced hydrophilic hydroxyl groups are not fully combined with the amino and carboxyl groups in proteins. 

Previous studies have shown that small amounts of polyphenols can promote the hydrophobicity of collagen membranes, while WCAs dropped even below the level of the uncrosslinked collagen membranes polyphenols were increased [18,44]. Polyphenols were mixed with collagen solutions to prepare crosslinked membranes, leading to complete contact between each other. In the presence of a certain amount of polyphenols, the hydrophilic hydroxyl groups were completely bound to the collagen, resulting in an increase in the hydrophobicity. As the amount of polyphenols further increased, the hydrophilicity of the membranes increased, with the number of unbound phenolic hydroxyl groups also increasing. In this study, the hydrophilicity of crosslinked AFSBs was increased by the low utilization of hydroxyl groups due to the inherent structure of AFSBs.

**Figure 5:**
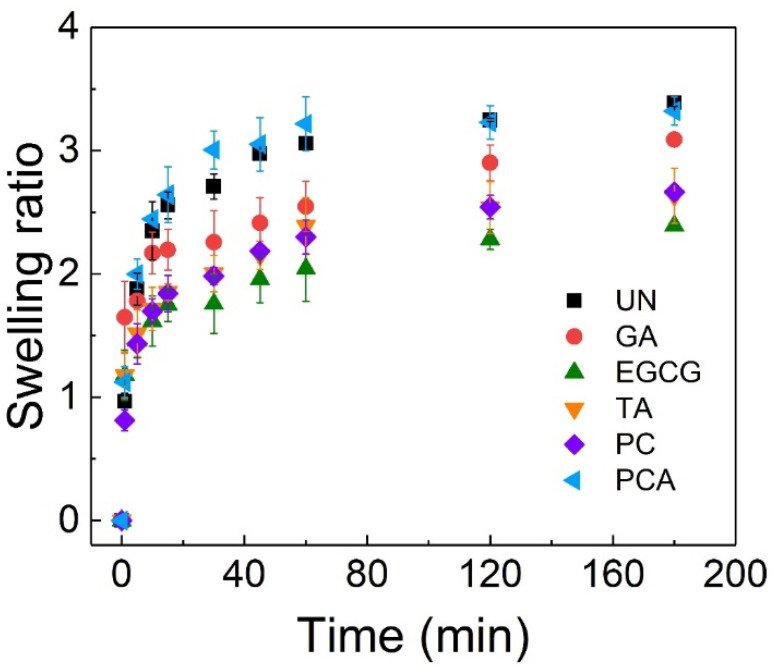
Swelling ratios of crosslinked AFSBs.

Figure 5 shows the swelling ratios of crosslinked AFSBs immersed in PBS for 3 h. The swelling ratios of samples increased rapidly in 30 min, and stabilized gradually across 3 h. The swelling ratios of UN and PCA were the largest, 3.39 and 3.32, respectively. The swelling ratio of PCA was faster than that of UN in the initial stage due to the better hydrophilicity of PCA. In addition, the equilibrium swelling ratios were reduced after crosslinking with GA (3.08), EGCG (2.39), TA (2.63) and PC (2.66), since the matrices of the AFSBs were tighter after crosslinking. Among them, the swelling ratio of EGCG was the lowest, showing the best crosslinking reinforcement of the fibers, which would enhance the stiffness of the matrix and avoid the loss of physical integrity and stability during the cell culture process. The swelling ratios can indicate the water uptake ability and crosslinking effect of the AFSBs, especially correlated with the mechanical properties [45]. The rapid swelling rate demonstrated the crosslinked AFSBs were able to rapidly absorb body fluids and wound exudate. This can provide a moist environment for the tissues and facilitate the transfer of cellular nutrients and metabolites.

### 3.3. Thermomechanical Properties

**Figure 6:**
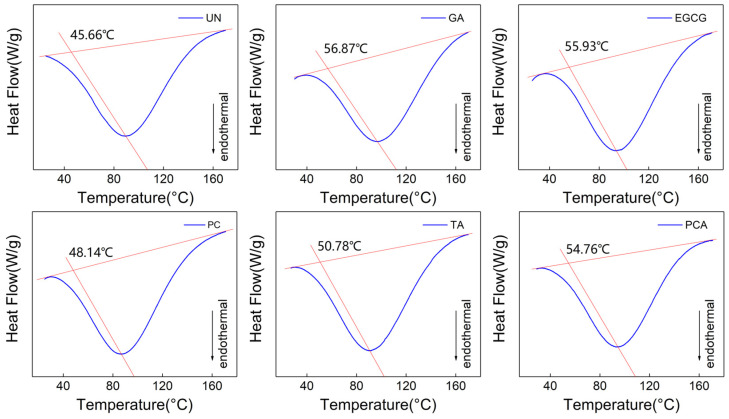
Differential scanning calorimetry (DSC) thermographs of AFSBs crosslinked by different polyphenols.

The thermal stability of crosslinked and uncrosslinked AFSBs was derived from differential scanning calorimetry (DSC). The typical DSC curves are presented in Figure 6, where T_d_ is the denaturation temperature of the samples, determined as the intersection of the baseline and the line owning the same slope as the tangent and through the peak point. The triple helix structure of collagen melts and dissociates into three randomly coiled peptide α-chains gradually with heating [46], and the endothermic peak is associated with the transformation from a triple helix to random coils of collagen [47].

The T_d_ of all the crosslinked AFSBs was increased compared to uncrosslinked AFSBs due to the interaction between the polyphenols and proteins. In particular, AFSBs crosslinked by EGCG (55.93 °C) and PCA (54.76 °C) had relatively high denaturation temperatures, which were increased by 10.27 °C and 9.1 °C compared with UN (45.66 °C). The enhancement of T_d_ in groups TA (50.78 °C) and PC (48.14 °C) can also be observed, but was not as effective as groups EGCG and PCA. The improvement of the thermal stability was related to the amount of H-bonds formed between the polyphenols and the collagen [18], since the stability of the triple helix configuration of collagen is conferred by the intermolecular H-bonds [48], and more H-bonds require more heat to dissociate. In addition, the T_d_ in groups EGCG and PCA was similar to that in group GA (56.87 °C) due to the fewer bonding sites of GA compared with polyphenols. Despite forming chemical bonds with amino groups of collagen side chains, GA can only react with free amino groups, while polyphenols can form H-bonds with hydroxyl, amino and amide groups. In this sense, polyphenols can improve the thermal stability of AFSBs through H-bonds, which was as effective as GA through chemical bonds. The improved thermal stability facilitates the preservation of the AFSBs as well as their further production.

Mechanical properties are vital in scaffold materials in order to cater for the demands of surgeries and combat stress incurred during in vivo implantation. The parameters of mechanical properties were studied and are summarized in Figure S2 and Table 1. The tensile strengths (TS) and elongation at break (EB) of the uncrosslinked AFSB were 2.6 MPa and 36.9%, respectively. After crosslinking, the TS and Young’s modulus (*E’*) of the AFSBs were enhanced in all groups. The AFSB crosslinked by EGCG showed the maximum TS of 8.55 Mpa, while the EB was 41.4%. The TS of polyphenol crosslinked AFSBs was intimately related to their crosslinking degree. Polyphenols tended to form H-bonds at multiple points to strengthen the intensity of individual fibers and the compactness of the matrix, thus enhancing mechanical strength. Tang et al. [49] proposed that the interaction strength between polyphenols and fibers was dependent on the number of galloyl groups, flexibility, molecular size, hydrophobicity in polyphenols and the molecular weight (polyphenols with large molecular weights can bind to proteins more effectively than polyphenols with small molecular weights) [49]. In consideration of the small molecular weight and only two hydroxyl groups in the PCA molecule, the ability of PCA to crosslink multiple fibers simultaneously was limited. The EGCG molecule was flexible with eight phenolic hydroxyl groups and can crosslink multiple fibers at the same time. As a result, the AFSB crosslinked by EGCG had the most superior mechanical properties. Another advantage of polyphenols in enhancing mechanical properties of AFSBs compared with GA is that polyphenols can crosslink with elastin, which is more responsible for recoil property at low stresses. As a result, the mechanical properties of the AFSB crosslinked by GA was not improved as much as those of polyphenols at the experimental concentration. This was consistent with the limited enhancement effect of GA on vascular extracellular matrices below 3.75 mg/mL demonstrated by Wang et al. [50]. The internal morphology of crosslinked AFSBs are presented in Figure S3. After crosslinking, the AFSBs maintained their intact porous substructure and well-organized fibers as the UN group, ensuring the exchange of nutrients and gases, and facilitating tissue regeneration.

### 3.4. In Vitro Enzymatic Stability and Biocompatibility

**Figure 7:**
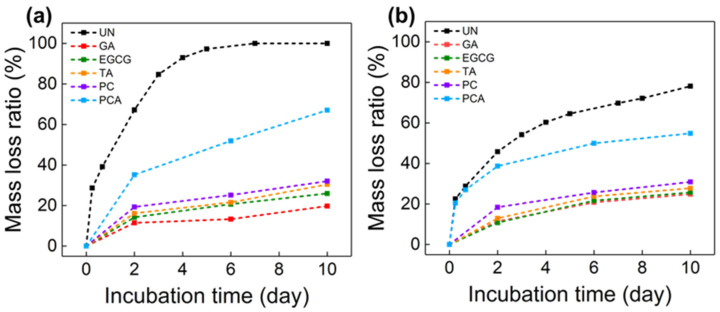
In vitro degradation of uncrosslinked AFSBs and polyphenol-crosslinked AFSBs. (**a**) Collagenase solution and (**b**) elastase solution.

It is well known that the limitations of ECM and collagen-based scaffolds are mainly due to their degradability in the wound environment by collagenase or elastase released at the site [51]. Therefore, the stability of collagenous biomaterials against degrading enzymes is essential for tissue remodeling. Polyphenols can form H-bonds with functional groups in main chain peptides, side chains of collagen and elastin to resist enzymatic degradation. As shown in Figure 7, the mass loss ratio (MLR) of all the crosslinked AFSBs was significantly lower than that of the uncrosslinked AFSBs after incubating with collagenase and elastase. The UN group was completely degraded after 7 days of incubation with collagenase, and degraded by 78% after incubation with elastase for 10 days. In the degradation experiments with collagenase, the MLR of the GA group was the lowest (24.8%) due to the high intensity of the amide bonds formed by GA and collagen. Among the polyphenol-crosslinking AFSBs, the ability against degradation of EGCG (25.7%) was the greatest, while that of the PCA group (54.8%) was the weakest. The same phenomenon occurred during the degradation by elastase; the limited improvement of the enzymatic stability in the PCA group was associated with the inferior crosslinking efficiency of the fibers in the AFSB. Specifically, the number of hydroxyl groups in one PCA molecule was too small to crosslink multi-fibers, which led to the inferior compactness and rigidity of the modified AFSBs, resulting in the weak anti-enzymatic degradation property of the PCA group. The result was consistent with a previous study that found that the OX-DAA group had a disappointing anti-enzymatic hydrolysis property despite having the highest degree of crosslinking [27]. It is worth noting that EGCG was comparable to GA in terms of the resistance to elastase, as GA only reacted with the e-amino, which is rare in elastin. The enzyme stability of the crosslinked AFSBs was proportional to the crosslinking ability of the polyphenols, since the degradation of the collagen initiate from the unwound regions in tropocollagen [52] and polyphenols tend to bond to the gap region rather than the overlap region of the collagen [53], which can affect the accessibility of collagenase to degradation sites. Briefly, polyphenols can improve anti-enzymatic degradation abilities through enhancing the stiffness of fibers, covering the binding site of enzymes on fibers and further protecting the fracture sites of enzymes from being occupied by enzymes. 

**Figure 8:**
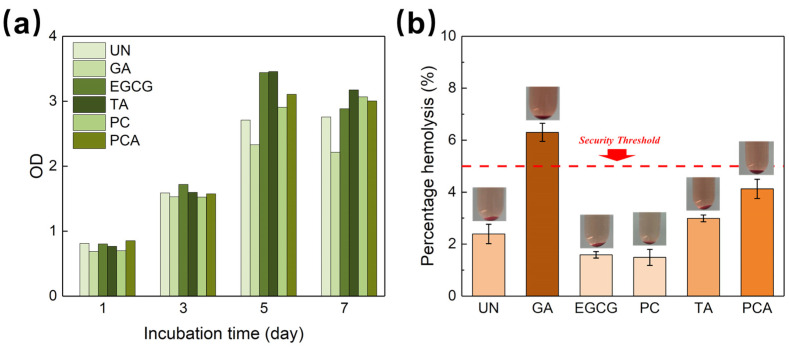
(**a**) Viability of fibroblasts (L929) and (**b**) the percentage hemolytic activity of uncrosslinked AFSBs and crosslinked AFSBs.

Figure 8a presents the viability of fibroblast (L929) cells cultured on AFSBs crosslinked by polyphenols, with the uncrosslinked AFSB serving as a control. It is obvious from the results that the polyphenol-crosslinked AFSBs showed no toxicity and excellent cell proliferation compared with the control group, while the AFSB crosslinked by GA had an inhibition on cell proliferation. Specifically, the optical density (OD) values were normalized against the control; on the fifth day, the viability of the TA group (128%) was comparable to the EGCG group (127%), and the viability of both the PC group (107%) and the PCA group (115%) were higher than that of the GA group (86%). The trace GA released with the slight degradation of AFSB during incubation can influence cell proliferation, whereas polyphenols do not. Wang et al. [50] revealed that GA can inhibit cell proliferation even at low concentrations, whereas PC was 100 times less cytotoxic to valve interstitial cells than GA. 

Observation confirmed that the presence of polyphenols greatly supported cell viability and proliferation. The enhanced cell proliferation might be attributed to the increased hydrophilicity of AFSBs crosslinked by polyphenols, which facilitated the adhesion and proliferation of cells. Moreover, the free radical scavenging properties of polyphenols can reduce the amount of reactive oxygen species (ROS) and provide a reductive environment for cell proliferation, since excessive amounts of ROS were damaging to biomolecules such as DNA, proteins and lipids, effecting proliferation [54]. Furthermore, the hemolysis percentages reveal the hemocompatibility of AFSBs, as shown in Figure 8b. The hemolysis percentage values were less than 5% for all polyphenol-crosslinking AFSBs, expect for the GA group, indicating compliance with the permissible level of hemolysis ratio for blood-contacting biomaterials required by the international standards. 

### 3.5. Microscopic Interactions between Polyphenols and AFSBs

**Figure 9:**
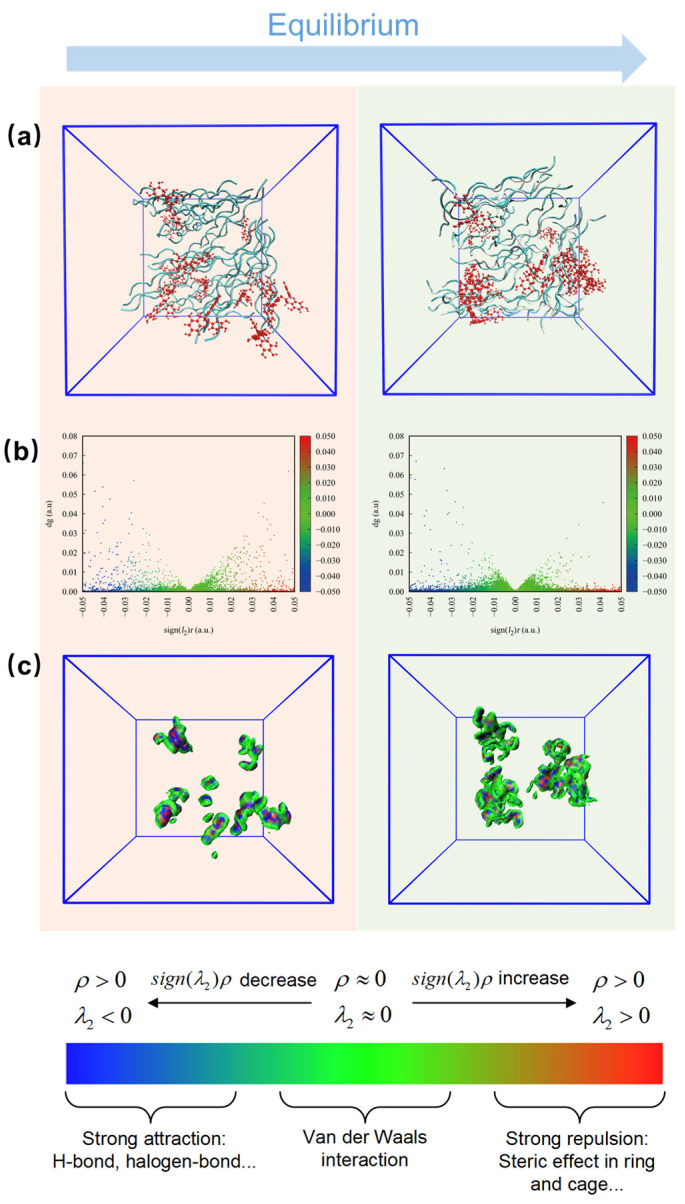
(**a**) Snapshots of final configurations; (**b**) independent gradient model (IGM) scatter diagram for the composite structures; and (**c**) *δ*_g_^inter^ iso-surfaces of EGCG system.

In order to further investigate the interactions between collagen and polyphenols at the microscopic level, the systems with collagen chains and polyphenol molecules were constructed and an MD simulation was carried out. The configurations of all systems are shown in Figure 9a and Figure S4, where collagen chains are in cyan and polyphenol molecules are in red. The initial collagen chains were fixed in the same position in each system, while the polyphenol molecules were inserted randomly. After equilibration, the polyphenol molecules tended to approach the collagen chains instead of aggregating by themselves and the collagen chains became more compact, indicating the intensive interactions between collagen and polyphenols. 

Additionally, an independent gradient model (IGM) [55] analysis was conducted to demonstrate the intermolecular interactions between collagen and polyphenols using the Multiwfn 3.8 software [56]. The occurrence region of non-covalent interaction is presented graphically through a non-covalent interaction (NCI) analysis [57]. The scatter diagram and iso-surface maps are shown in Figure 9b,c and Figure S4. The regions of sign(*λ*) *ρ* > 0 a.u. and sign(*λ*) *ρ* < 0 a.u. stand for the intermolecular repulsion and attraction effects in all systems, where the strong attraction, van der Waals (vdW) and strong repulsion are exhibited with blue, green and red, respectively. *δ*_g_ is a critical descriptor that is positively correlated with the intensity of the molecular interaction. It can be seen clearly that both the intensity of the H-bonds and the vdW force were increased after equilibrium, attributed to the attraction of polyphenols to collagen chains in all systems. Meanwhile, the molecular attraction effect was stronger than the repulsion effect, suggesting that the interactions between collagen and polyphenols are favorable and spontaneous among all systems. Among them, the repulsion effect of PCA system was the strongest, partially counteracting the H-bonds effect between collagen and PCA. Furthermore, the 3D iso-surfaces at *δ*_g_ = 0.005 a.u. are depicted to show more details about the configurations of collagen-polyphenols complexes. The interaction region increases with the crosslinking of collagen and polyphenols. For the EGCG, PC and TA systems, the regions were concentrated and continuous, owing to the multiple phenolic hydroxyl groups in their molecules compared to the PCA system. 

**Figure 10:**
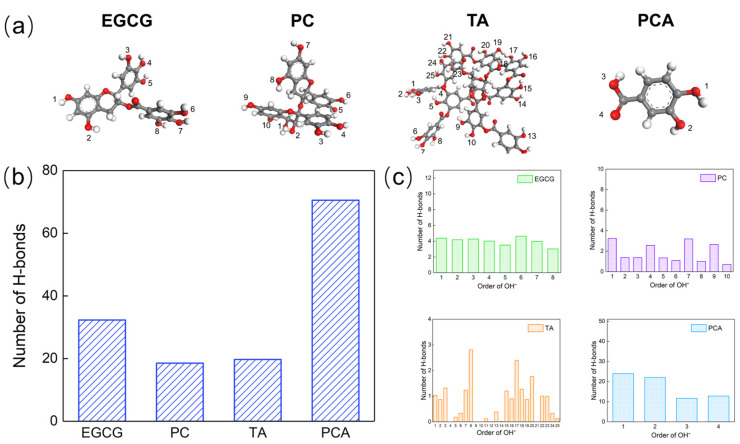
(**a**) The optimized molecule structure of polyphenols (Each hydroxyl group in the polyphenol molecules and all O atoms in the PCA molecules were numbered); (**b**) the total number of hydrogen bonds (H-bonds) formed between collagen and polyphenols; and (**c**) the number of H-bonds formed between collagen and each identified hydroxyl group in polyphenols.

Considering the importance of H-bonds in crosslinking between AFSBs and polyphenols, as well as the limitations of H-bonds analysis through experiments, MD simulations were performed in order to analyze the number and characteristics of H-bonds in complex systems. The structures of polyphenol molecules are shown in Figure 10, where each hydroxyl group in the polyphenols molecules was assigned with a serial number. The number of hydroxyl groups in EGCG, PC and TA are 8, 10 and 25, respectively. Although a PCA molecule has two hydroxyl groups, the oxygen atoms in the -COOH group were also assigned, as they can also form H-bonds with collagen.

Figure 10 shows the total number of H-bonds formed between collagen and polyphenols, which is only related to the accessible acceptors and donors. The number of H-bonds in the PCA system (70.5) was the highest, followed by EGCG (32.2), TA (19.708) and PC (18.5). Although the number of hydroxyl groups was controlled to be the same in each system, the -COOH group in PCA was also able to form H-bonds with collagen, which increased the number of H-bonds. Fibers in AFSBs had a microstructure that limited their contact with larger polyphenol molecules; miniature molecules such as EGCG and PCA were more likely to cross the fibrous gaps and formed H-bonds with collagen chains. Moreover, the ability of hydroxyl groups in polyphenol molecules to form H-bonds was a major factor. Some hydroxyl groups were limited in forming H-bonds due to the spatial resistance of the functional groups within the molecules; the number of hydroxyl groups that were able to form H-bonds was much smaller than that possessed by a polyphenol molecule.

**Figure 11:**
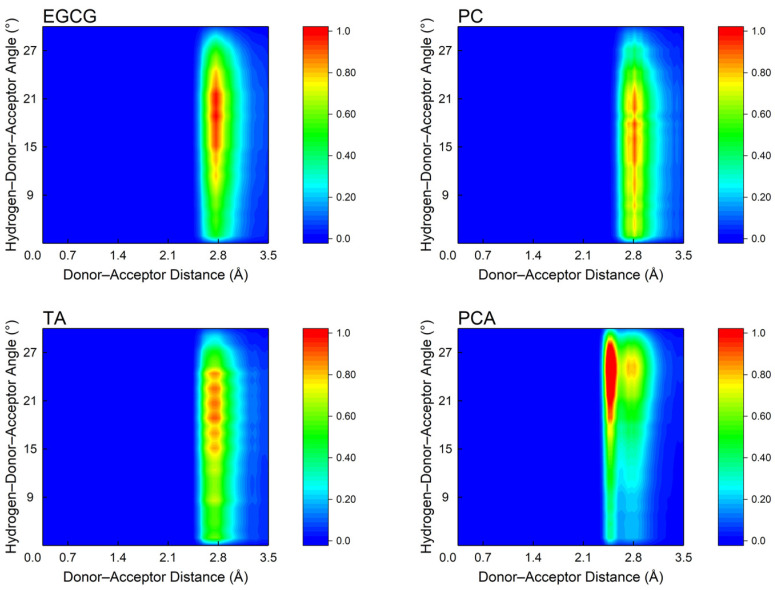
Distance and angle distribution of H-bonds formed between collagen and polyphenols.

Figure 11 shows the distance and angle distributions of H-bonds in all systems. There was a higher probability for the distance and angle of H-bonds within 0.24~0.3 nm and 10~30°, confirming the existence of strong H-bonds. Specifically, the angle distribution region of H-bonds in the EGCG system was lower than in other systems, showing that the H-bonds in the EGCG system were stronger. In addition, the distribution of H-bonds in the TA and PC systems was not concentrated, which might be related to the structural rigidity of the TA and PC molecules, and the significant differences in the reactivity of each hydroxyl group.

As mentioned previously, the number of H-bonds formed by polyphenols is related to the ability of hydroxyl groups to participate in the forming of H-bonds, as was explored and shown in Figure 10c. Apart from the hydroxyl groups, the -COOH group contributed an additional 24.5 H-bonds with collagen in the PCA group. Meanwhile, the PCA molecule is small and simple with a strong availability of hydroxyl groups, which facilitated the diffusion of PCA into the fibers and the formation of H-bonds with collagen. Each hydroxyl group in EGCG had the same reactivity and the opportunity to form H-bonds with collagen. The bonding capacity of each hydroxyl group in PC was diverse and can be affected by adjacent hydroxyl groups; only one hydroxyl group in each molecular region had the highest bonding capacity. Although a TA molecule had 25 hydroxyl groups, some hydroxyl groups were embedded inside the molecule due to the large size of the molecule and the high potential resistance, and therefore, they were unable to come into contact with collagen. The terminal hydroxyl groups of the TA molecule had a more powerful bonding ability than the internal hydroxyl groups.

The size and structure of polyphenol molecules resulted in the different accessibility of hydroxyl groups to crosslink fibers in AFSBs with specific microstructure, presenting a different number of H-bonds formed with collagen. The number of H-bonds contributed to the thermal stability of the AFSBs, since the stability of the triple helix was determined by intermolecular forces that can be enhanced by H-bonds regardless of position, and the improvement in hydrophilicity was only correlated to the ratio of consumed hydroxyl groups to those introduced. It is worth noting that the mechanical properties and resistance to degradation of crosslinked AFSBs were not completely positively correlated with the number of H-bonds. Polyphenols with multiple crosslinking sites were more effective in reinforcing fibers. In addition, the inhibition of enzymatic activity by polyphenols also affected the resistance to enzymatic degradation of crosslinked AFSBs. Among these polyphenols, EGCG had a relatively low molecular weight, multiple phenolic hydroxyl groups and a high accessibility and reactivity to each hydroxyl group. Generally, the properties of AFSB crosslinked by EGCG were the most superior under a certain concentration of hydroxyl groups.

**Figure 12:**
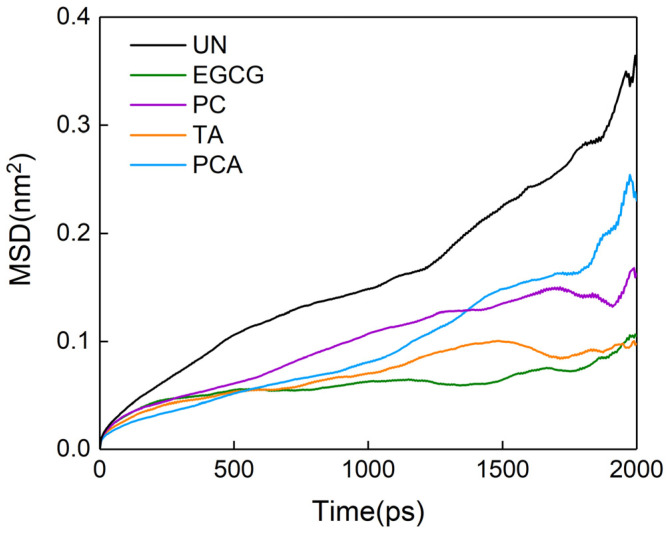
Mean square displacement (MSD) of collagen chains in different polyphenol systems.

The mean square displacement (MSD) curves of collagen in different systems are shown in Figure 12, and the self-diffusion coefficient was obtained from the slope of the MSD curve in order to assess the effect of the interaction between collagen and polyphenols on the fluidity of collagen chains. The self-diffusion coefficients of collagen in the systems with polyphenols were lower than that in the system without polyphenols, indicating that the fluidity of collagen was decreased after introducing polyphenols. Collagen crosslinked with EGCG showed the lowest fluidity, whereas PCA showed the largest, confirming the optimal mechanical properties of group EGCG. The decreased fluidity of collagen acted to reinforce the collagen fibers, compacting the matrix.

## 4. Conclusions

In this work, AFSBs were crosslinked with polyphenols (i.e., EGCG, PCs, TA and PCA) in order to improve their thermal stability, enzymatic stability and mechanical properties. The influences of polyphenols on the properties of crosslinked AFSBs were explored through experiments and MD simulations. Crosslinking efficiency is associated with the amount of H-bonds formed by polyphenols, which is related to their molecular weight, molecular size, number and the position of hydroxyl groups. Polyphenols with a simple molecular structure and low molecular weight (such as PCA and EGCG) are more effective in improving the thermal stability of AFSBs by up to 10.27 °C; however, they have limited enhancement on hydrophilicity. Polyphenols with multiple phenolic hydroxyl groups and low potential resistance (such as EGCG) are more favorable for the mechanical properties and enzymatic stability of AFSBs, since the high participation ability of each hydroxyl group leads to an enhanced degree of crosslinking. EGCG has superior enzymatic stability and mechanical properties compared to GA, as well as excellent biocompatibility and integrity of the collagen triple helix. Hopefully, the conclusions reached by this study can provide a potential strategy for improving the clinical performance of ECM materials, as well as a deeper understanding of the interactions between polyphenols and ECM materials.

## Figures and Tables

**Table 1:** Mechanical properties of uncrosslinked and crosslinked AFSBs.

Sample	Young’s Modulus (MPa)	Ultimate Tensile Strength (MPa)	Elongation at Break (%)
UN	9.68	2.60	36.9
GA	12.38	4.35	46.9
EGCG	30.62	8.55	41.4
PC	27.51	7.03	43.9
TA	27.65	7.34	43.4
PCA	17.56	3.61	30.0

## Data Availability

The data presented in this study are available on request from the corresponding author (due to privacy).

## Supplementary Materials

The following supporting information can be downloaded at: https://www.mdpi.com/article/10.3390/polym16081111/s1. Table S1. Different compositions of collagen/polyphenols systems in molecular dynamic (MD) simulations. Figure S1. Ninhydrin assay images of crosslinked AFSBs. Figure S2. Stress–strain curves of uncrosslinked and crosslinked acellular fish swim bladders (AFSBs). Figure S3. Scanning electron microscopy (SEM) micrographs of uncrosslinked and crosslinked AFSBs. Figure S4. (a) Snapshots of final configurations, (b) Independent gradient model (IGM) scatter diagram for the composite structures and (c) *δ*_g_^inter^ iso-surfaces of EGCG system. Refs. [23,58] are cited in the Supplementary Materials.
