# Supplementary material for: Influences of Polyphenols on the Properties of Crosslinked Acellular Fish Swim Bladders: Experiments and Molecular Dynamic Simulations
Source: Polymers (Basel). 2024 Apr 16;16(8):1111. doi: 10.3390/polym16081111 (PMC11054729; doi:10.3390/polym16081111)
Supplement: Supplementary file 1 [file polymers-16-01111-s001.zip › polymers-2930730-supplementary.pdf]

Supporting Information

for

Influences of Polyphenols on the Properties of Crosslinked Acellular Fish Swim Bladders: Experiments and Molecular Dynamic Simulations

Yuqing Han[1], Jie Jiang[1], Jinjin Li[1*], Ling Zhao[1,2] and Zhenhao Xi[1,2*]

[1] State Key Laboratory of Chemical Engineering, School of Chemical Engineering, East China University of Science and Technology, Shanghai 200237, China

[2] Shanghai Key Laboratory of Multiphase Materials Chemical Engineering, East China University of Science and Technology, Shanghai 200237, China

*Corresponding authors:

E-mail (Jinjin Li): lijinjin@ecust.edu.cn

E-mail (Zhenhao Xi): zhhxi@ecust.edu.cn

Postal Address: Campus Box 369, No. 130 Meilong Road, Shanghai 200237

Characterizations

ATR-FTIR spectra measurements

Attenuated total reflection flourier transformed infrared (ATR-FTIR) spectra were obtained through a Nicolet is50 spectrometer (Thermo Fisher Scientific, USA) with a diamond crystal detector. Samples were sliced into squares of 8 mm × 8 mm and positioned over the diamond crystal on the ATR apparatus. During the experiment, standardized pressure was applied to maintain the integrity of samples and ensure optimal optical contact between the samples and the crystal. Before each acquisition, the background spectrum was subtracted and a single spectrum was collected for each sample. All spectra were recorded with a resolution of 4 cm^{-1} with 32 scans at the wavelength range of 600 ~ 4000 cm^{-1}.

Differential scanning calorimetry measurements

The thermal stability of acellular fish swim bladders (AFSBs) before and after crosslinking was determined through differential scanning calorimetry (DSC; DSC204HP, Netzsch, Germany). The maximum temperature at which the stability of internal triple helix structures can maintain under gradual heating was defined as the denaturation temperature. Briefly, approximately 5 mg dried sample was packed in a DSC aluminum crucible with an empty crucible as a reference. The test temperature was set from 20 to 180 °C with a constant heating rate of 5 °C/min in a nitrogen atmosphere.

Crosslinking degree

The crosslinking degree of AFSBs was obtained by the ninhydrin assay[1]. Briefly, 0.1 mg ninhydrin was dissolve in 4 mL ethanol and added to about 6 mg samples. Then the solution was heated in an oven at 100 °C for 30 mins. After the solution was cooled to room temperature, 5 mL of 50% isopropanol solution was added to elute. The optical absorbance of the supernatant was measured at 570 nm by a Microplate Reader, and the crosslinking degree was calculated as follows:

$$Crosslink\ degree\ (\%) = (OD_c - OD_s)\ /\ OD_c \times 100\%$$

Where OD_c is the absorbance value of the uncrosslinked AFSB and the OD_s represents the absorbance value of the crosslinked AFSB.

Water contact angle tests

The hydrophilicity of AFSBs was determined by the water contact angle (WCA) at room temperature through a goniometer (JC2000A, Shanghai Zhongcheng Digital Equipment Co., China). 2 μL de-ionized (DI) water was deposited carefully on the surface of the sample, the angle between the surface and the water drop was measured every regular time. Each data was obtained from the average of three positions on the corresponding AFSB.

Swelling behavior

To analyze the fluid absorption capacity and compactness of the crosslinked AFSBs, the swelling behaviors were detected by immersing the samples in phosphate-buffered

saline (PBS). Initially, the freeze-dried samples were weighted (W_i), and then immersed in PBS for 3 h. At each specific time, the samples were removed and wiped off with filter papers carefully. The wet samples were weighted and recorded as W_t. The swelling ratio was calculated as follows:

$$Swelling\ ratio = (W_t - W_i) / W_i$$

Mechanical tests

Mechanical characters were performed by a universal testing machine (AG-2000A, Shimadzu, Japan). AFSBs were sliced into rectangular shapes with 25 mm in length and 4 mm in width and utilized in hydrated conditions after soaking in PBS for 12 h. The uniaxial tensile test was conducted at a test speed of 2 mm/min with 3 samples and increased tension until failure. The tensile strength (TS), elongation at break (EB) and Young's modulus ($E^{'}$) of the AFSBs were determined based on the cross-sectional area of the samples. Specially, the $E^{'}$ was determined using the slope of the second linear region of the tensile stress - strain curve[2]. Apart from this, the section morphology of the crosslinked AFSBs were analyzed through a JSM-6360LV (JEOL Ltd., Tokyo, Japan) scanning electron microscopy (SEM).

In *vitro* enzymatic stability

The ability of polyphenols to inhibit enzymatic activity and the enzymatic stability of crosslinked AFSBs were explored in both collagenase and elastase solution. About 10 mg freeze-dried samples was incubated in 1 mL collagenase solution (50 units/mL, Shanghai Yuanye Bio-Technology Co., China) and 1 mL elastase solution (45 units/mL,

Shanghai Yuanye Bio-Technology Co., China) at 37 °C for 10 days, respectively. The AFSBs were collected every regular time, washed with DI water completely, then lyophilized again and weighed. The mass loss ratio (MLR) was calculated as follows:

$$MLR\ (\%) = \frac{mass\ before\ degradation - mass\ after\ degradation}{mass\ before\ degradation} \times 100\%$$

In *vitro* biocompatibility

In vitro cell proliferation. The fibroblasts (L929) were cultured onto the surfaces of AFSBs to evaluate the biocompatibility of the modified AFSBs. Briefly, AFSBs were cut into 8 mm × 8 mm squares, immersed in 75% (v/v) ethanol for 2 h and irradiated with UV light overnight for sterilization. Subsequently, the samples were freeze-dried and placed at the bottom of a 48-well plate with stainless steel rings on top to prevent samples from floating. 30 μL Dulbecco's modified Eagle's medium (DMEM) with 10 000 L929 were inoculated onto the surfaces of the AFSBs and maintained in an incubator for 2 h to allow cell adhesion. An additional 250 μL DMEM was added into each well and the plate was incubated for 7 days. The CCK-8 test was performed each two days according to the description. 10% CCK-8 reagent in DMEM was prepared and added to the wells to incubate 2 h. Optical density (OD) values were obtained by measuring the supernatant of each well through a microplate reader (SPECTRAmax384, Molecular Devices, USA) at 450 nm.

Hemolytic activity. The hemolytic activity of each crosslinked AFSB was determined by incubating the samples with the erythrocyte suspension. Briefly, the pure erythrocyte suspension was obtained by centrifugating the porcine whole blood (H23688, Shanghai

Yuanye Bio-Technology Co., China) and washed with PBS at pH 7.4. The crosslinked AFSBs were added to the porcine whole blood diluted in PBS, while the red blood cells (RBCs) mixed with DI water was used as a positive control and PBS was used as a negative control. After 1 h incubation, the supernatants were collected by centrifugating the samples for 5 min and measured at 540 nm to determine the toxicity on RBCs. The hemolysis percentage was calculated as $(OD_s - OD_n) / (OD_p - OD_n) \times 100\%$, where OD_s represents sample absorbance, OD_p and OD_n are denoted as positive and negative control absorbance, respectively.

Simulation details

Table S1. Different compositions of collagen/polyphenols systems in molecular dynamic (MD) simulations

Case	System	Number of Collagen	Number of H_2O	Number of Polyphenols	Number of hydroxyl groups
1	UN	10	6000	-	-
2	EGCG	10	6000	13	104
3	PC	10	6000	10	100
4	TA	10	6000	4	100
5	PCA	10	6000	51	102

Results and Discussion

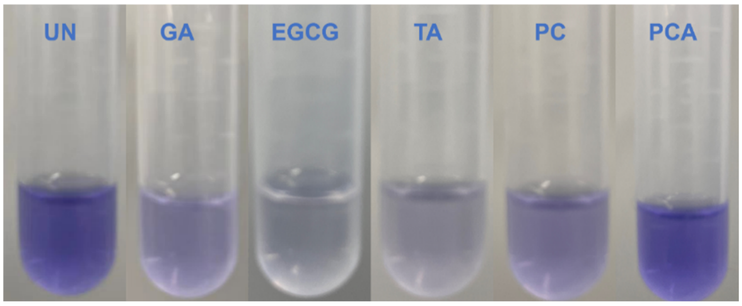

Figure S1. Ninhydrin assay images of crosslinked AFSBs

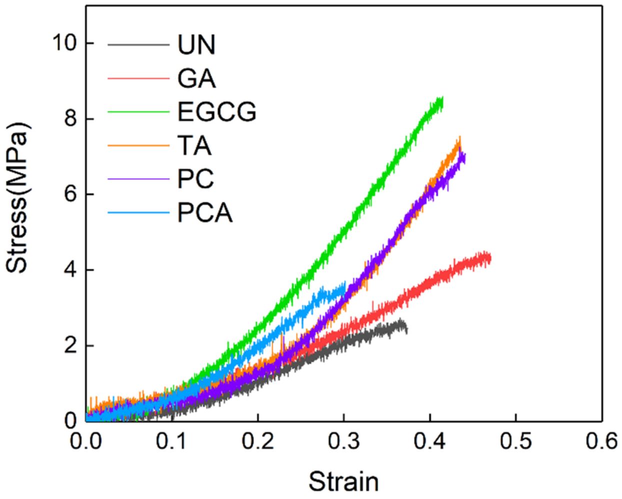

Figure S2. Stress–strain curves of uncrosslinked and crosslinked acellular fish swim bladders (AFSBs)

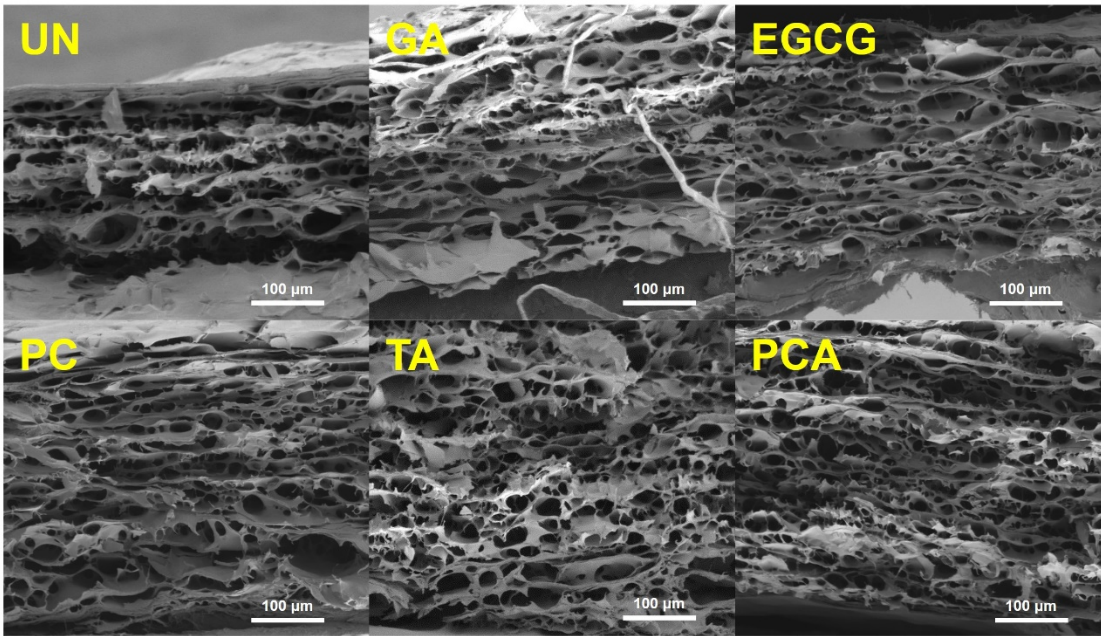

Figure S3. Scanning electron microscopy (SEM) micrographs of uncrosslinked and crosslinked AFSBs

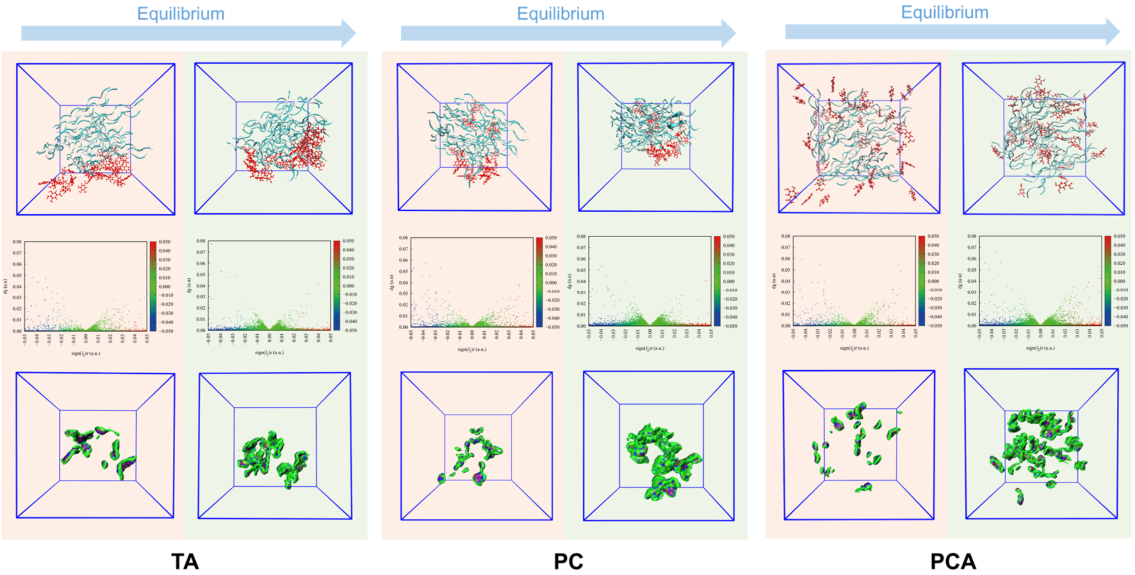

Figure S4. (a) Snapshots of final configurations, (b) Independent gradient model (IGM) scatter diagram for the composite structures and (c) δg^{inter} iso-surfaces of EGCG system

References

1. Kaparekar, P. S.; Pathmanapan, S.; Anandasadagopan, S. K. Polymeric scaffold of Gallic acid loaded chitosan nanoparticles infused with collagen-fibrin for wound dressing application. *Int. J. Biol. Macromol.* **2020**, *165*, 930-947.
2. Liu, J.; Li, B. H.; Jing, H. M.; Qin, Y. B.; Wu, Y. J.; Kong, D. L.; Leng, X. G.; Wang, Z. H. Curcumin-crosslinked acellular bovine pericardium for the application of calcification inhibition heart valves. *Biomed. Mater.* **2020**, *15* (4), 045002.
